# The Global Human Monkeypox Outbreak and Management: A Comprehensive Literature Review

**DOI:** 10.7759/cureus.32557

**Published:** 2022-12-15

**Authors:** Ibrahim M Dighriri, Shafiqah H Braiji, Malek M AlAnazi, Mona J Ayyashi, Aisha A Khubrani, Yasir B Khormi, Lujain A Shbeir, Sarah I Alatif, Aisha E Alfagih

**Affiliations:** 1 Department of Pharmacy, King Abdulaziz Specialist Hospital, Taif, SAU; 2 Department of Pharmacy, East Jeddah Hospital, Jeddah, SAU; 3 Department of Pharmacy, Dr. Mohammed Alfagih Hospitals, Riyadh, SAU; 4 Department of Pharmacy, Community Pharmacy, Riyadh, SAU; 5 Department of Pharmacy, Community Pharmacy, Jazan, SAU; 6 Faculty of Pharmacy, Jazan University, Jazan, SAU; 7 Faculty of Pharmacy, Princess Nourah Bint Abdulrahman University, Riyadh, SAU; 8 Faculty of Pharmacy, King Khalid University, Abha, SAU; 9 Faculty of Medicine, Umm Al-Qura University, Qunfudhah, SAU

**Keywords:** monkeypox prevention, monkeypox treatment, outbreak, epidemiology, monkeypox virus

## Abstract

Monkeypox (MPX) belongs to the genus Orthopoxvirus (OPV), family Poxviridae, and sub-family Chordopoxvirinae. Human monkeypox (HMPX) is a viral zoonotic illness caused by the monkeypox virus (MPXV). Several non-endemic countries have confirmed MPX cases across the globe. Therefore, consider an outbreak to be a global health emergency. MPXV transmits from animals to humans via infected animals, and there is currently human-to-human transmission, notably among guys who have sexual relations with males. Healthcare interventions are required to stop outbreaks. These include strict isolation and care for MPX patients while they are still contagious or until the skin lesions dry out and crust over. JYNNEOS was approved as a vaccine for the prevention of MPXV. Tecovirimat is licensed to treat severe MPX or risk developing a serious disease. We should encourage international cooperation to conduct clinical trials investigating the effectiveness and safety of MPXV vaccines and antiviral medications. Precautions must be taken at the global level to prevent an MPXV outbreak.

## Introduction and background

Human monkeypox (HMPX) is a zoonotic viral disease caused by the monkeypox virus (MPXV), which infects conditions that are part of the Orthopoxvirus (OPV) species in the Poxviridae family [1]. The Poxviridae family includes the Variola virus, which causes smallpox, the vaccinia virus, and cowpox [2]. Monkeypox (MPX) was identified in 1958 when two epidemics of pox-like disease occurred in study colonies of monkeys [3]. When monkeys transferred from Singapore to a Danish research center were sick, the MPXV was distinguished in 1959 [4]. The first human cases were discovered in the Democratic Republic of the Congo in 1970, during increased efforts to eradicate smallpox [5]. HMPX was identified in 1987 as the most significant OPV that affects humans [1]. HMPX has been documented in people from Africa since then. It is worth noting that MPX is prevalent in several African nations and can kill up to one out of every 10 people who develop the disease. Instances of MPX in humans have emerged outside Africa and have been linked to foreign travel or imported animals, leading to cases in Europe, the United States, and Asia [6].

Vaccination against MPXV has previously resulted in coincidental immunity; however, eradication of smallpox and the subsequent absence of vaccination attempts allowed MPX to attain therapeutic importance [7]. Moreover, since the majority of cases of MPX occur in Africa, possible misreporting may lead to an underestimating of pathogen harm [8]. HMPX infects several types of mammals. However, its native host reservoir is unclear [9,10]. Transmission is believed to occur through saliva, touching fluid or surface particles, or respiratory excretions. Another route of viral exposure could be viral shedding through feces [11,12].

HMPX has features that closely resemble smallpox in terms of the appearance of symptoms, rash pattern, and incidence of dermatitis. However, it is less severe than smallpox, with a reduced death rate [9]. Lymph node swelling that begins early, often at the commencement of fever, distinguishes MPX from smallpox. A rash generally emerges one to three days after beginning fever and lymphadenitis, with lesions emerging simultaneously and developing at the same pace. Their dispersion is primarily peripheral, but with severe disease, they may cover the entire body. The infection may linger up to four weeks before the area heals [2,13]. Understanding the HMPX virus outbreak and its associated complications will improve attempts to find ways to prevent attacks and protect humans [14]. As result, this study provides a comprehensive review of the literature on HMPX outbreaks and management.

## Review

History and outbreak of HMPX

In 1970, a nine-month-old child was taken to Basankusu Hospital for likely smallpox, and the first HMPX case was documented [5]. The child's sample was sent to the World Health Organization (WHO), where viral isolation indicated HMPX [15]. The WHO then documented 20 human cases between 1972 and 1976 [16,17]. After that, 15 more cases were reported in 1978 [18]. However, between 1970 and 1979, the WHO documented 54 cases [19,20]. Arita et al. documented 98 cases post-1980 [21]. Between 1981 and 1983, Jezek et al. recorded 132 instances [22]. A total of 350 patients were identified between 1980 and 1986 [20]. From 1993 and 1995, no cases of HMPX were documented. However, a sustained outbreak happened in 1996 and 1997 [23].

The first case was in February 1996. However, the outbreak was not recognized until the end of July, when additional persons were infected [24]. Between 1996 and 1997, 511 cases were reported in 54 communities in the Katako-Kombe zone and 24 in the Lodja zone [25]. This outbreak is the most significant cluster of probable cases ever documented, spanning a broad region of the Katako-Kombe and Lodja zones. This could be attributed to the local population's decreased antibody protection due to the termination of smallpox immunization [26]. In 2001, four cases of HMPX were recorded in the Mbomou area [27]. Between 1998 and 2002, around 1265 cases were detected [28].

In 2003, the first outbreak of HMPX outside Africa occurred in the United States, with 37 confirmed cases [6,29]. Unlike HMPX, most cases in Africa involved children, while most cases in the United States involved adults [30]. In Unity State, in 2005, 10 confirmed, 9 probable, and 30 suspected HMPX cases were recorded [31]. Person-to-person infection was documented for up to five generations, yet no fatalities occurred during this episode [31,32]. The MPXV strain discovered during this crisis was thought to have a unique genetic structural variant associated with the Congo Basin [32]. From 2005 to 2007, 760 cases of HMPX were found in 9 areas [33]. In 2010, an infection in the Likouala area resulted in 10 cases, 2 of which were confirmed and 8 of which were suspected [34]. In 2010, two HMPX cases were verified in Africa. The tested strain was linked to the incidence in 2001 [34]. Between 2010 and 2014, incidents reached more than 2,000 cases [35].

From 2015 to 2016, at least twelve people were infected with HMPX; three died [36,37]. In 2017, the Likouala region announced an outbreak of HMPX with 88 cases, 7 confirmed, and 6 deaths [38]. On March 13, the Congo government publicly declared the outbreak. Children <15 years were most affected [38]. From 2017 to 2018, 101 confirmed cases were reported in 25 states [39]. Several isolated occurrences of the disease have been documented on several continents where it is not found naturally, including Europe and Asia. The current epidemic is worldwide and spreads from person to person. The cases have increased dramatically from May to November 20, 2022, which is 80,328 cases infected by MPXV in 110 different locations around the world and the deaths of 53 people in 15 countries [40,41].

MPX classification

MPXV is a member of the Poxviridae family, which includes smallpox, vaccinia, and cowpox viruses. Poxviruses are the most important known vertebrate viruses that affect humans, other vertebrates, and arthropods. Poxviruses are classified into 28 genera and sub-families: Chordopoxvirinae and Entomopoxvirinae [42]. A linear double-stranded deoxyribonucleic acid (dsDNA) genome and enzymes that create messenger ribonucleic acid (mRNA) are found in virions. MPXV replicates in the cytoplasm for infected cells [43]. Chordopoxvirinae is made up of around 10 genera. Camelpox, MPXV, variola, cowpox, ectromelia, Racoonpox, and other viruses belong to the genus OPV [44]. MPXV is a rodent virus whose detection is based on biological characteristics and viral deoxyribonucleic acid (DNA) endonuclease sequences [44]. There is some genetic variation among MPXV obtained from West and Central Africa. Genome analyses have provided substantial evidence that MPXV is unrelated to the variola virus. Significant attempts were undertaken in the pre-molecular period to differentiate the viruses using serological responses. These were sensitive experiments since the viruses shared most antigens [45].

MPX etiology

MPXV can infect many small animals, making it hard to control. The largest animal reservoirs for viruses are rodents like squirrels and rats, which hunted for food [35]. Environmental changes have made people more aware of animals. Climate change, deforestation, urbanization, cross-border migration, poverty, unsafe traditional practices, and civil wars are reasons why MPXV is returning. Underreporting and under-recognition of cases, lack of access to healthcare facilities, inexperienced staff, and poor laboratory diagnosis would help an epidemic spread [7]. Reported cases have been among gay and bisexual men aged 20-50, but it's unclear if this is because of their sexual habits or if it's just a coincidence [46].

Reservoir of MPX

MPXV has not yet been isolated from Cercocebus atys and Funisciurus anerythrus, so its host reservoir is unknown [47,48]. MPXV was transmitted from animals to humans. Mice, monkeys, primates, hedgehogs, squirrels, pigs, and rats are animal reservoirs in African areas where MPXV was previously widely recorded [49].

MPX transmission mode

MPXV is often spread from animals to people by bites from contaminated animals, through blood or bodily fluids, or by consuming inadequately cooked infected animals [50,51]. Following smallpox clearance, the population's antibody against OPV gradually deteriorates. Infrequent human-to-human infection of MPXV occurs primarily through direct face-to-face contact or a significant quantity of droplets in the air [49,52]. It may also be passed from mother to child during pregnancy or by fluids from an infected person or virus-contaminated items such as clothes and bedding. Furthermore, sexual transmission is possible [49,52,53].

The majority of cases in the latest epidemic were among young men who had sex with men who had genital sores, which could constitute intimate contact [6]. People who haven't been immunized against smallpox are more vulnerable to MPXV. Workers who slaughter wild game, pet owners, personnel at animal breeding facilities, and direct connections may be at increased risk [53].

MPX pathogenesis

MPXV enters the host, multiplies at the entrance point, and circulates through the lymphatic system after being transferred by an animal or a human. This results in bloodstream infections, which are referred to as primary viremia. The pathogen then starts to multiply in lymphoid tissues and distal lymph nodes, infecting the epithelium and tertiary organs, and causing mucosal and skin ulcers. The incubation phase typically lasts one to two weeks, with a maximum of three weeks [54].

Clinical characteristics and differential diagnosis of MPX

The clinical manifestations of MPX are similar to those of smallpox. Over 90% of MPX patients begin with fever, dermatitis, headaches, and respiratory complaints, and over 80% have lymphadenitis, oral ulceration, and diarrhea [49].

Smallpox, chickenpox, generalized varicella, disseminated zoster, disseminated herpes simplex, and eczema herpeticum are the most common differential diagnoses for MPX. The main difference between MPX and smallpox is that MPX causes lymphadenopathy, whereas smallpox does not. Also, MPX symptoms aren't as bad as smallpox symptoms. But both infections take the same amount of time to spread, begin showing signs, and end. Also, the pattern of skin lesions is like a wheel, and the eruptions are the same in both conditions [55].

MPX takes one to two days for each stage of skin eruptions and even five to seven days for the pustular phase while chickenpox rashes go from macules to crusts quickly, in just one day. So, MPX skin lesions last much longer than those from chicken pox [56]. Chickenpox is contagious until the last scabs harden, which can take up to three weeks. MPX, on the other hand, could spread for weeks or even months. Also, MPX takes much longer to show symptoms than chickenpox, so the person who first gets it can spread it to many more people before anyone notices. Other factors differentiating MPX from varicella were the high-grade fever that preceded it and lymphadenopathy in MPX [40,56].

In addition, MPX infections are more likely to affect the palms and soles than chickenpox infections. Epidemiological patterns can also help determine the difference between the two infections. MPX is a zoonosis, which means it comes from animals and can be spread from animals to humans or from humans to humans. On the other hand, chickenpox only comes from humans and is spread only between humans. Furthermore, chickenpox is much more likely to cause a second infection than MPX. Varicella mainly affects young children while adults are likely to get MPX [57,58].

MPX diagnosis

The rapid detection of MPXV is critical to minimize its transmission, but clinical signs alone are insufficient to prove it. As an aspect of the confirmation procedure, laboratory techniques, such as microscopic examination, immunologic testing, histology, and molecular approaches, should be employed. Each situation's intricacy, time commitment, and resources dictate the strategy used [9].

Using Electronic Microscope

Under an electron microscope, MPXV appears intracytoplasmic, like a brick, with lateral bodies and a central core. This method does not offer a definitive diagnosis since OPV species cannot be differentiated morphologically. However, it does show that the virus is a member of the Poxviridae family [59,60]. In addition, the electron microscope's sensitivity is limited, and the preparation of the electronic sample is difficult and time-consuming. Meanwhile, electron microscopy is expensive and very complex, limiting its practical detection applications. MPXV was observed in Vero/hSLAM cells isolated from the hand and abdomen using thin-section electron microscopy [61,62].

Immunological Methods

Using enzyme-linked immunosorbent assays (ELISA) to identify immunoglobulin G (IgG) and immunoglobulin M (IgM) antibodies and immunohistochemistry to detect viral antigens [49]. Antiviral antibodies and T-cell responses have increased around the start of sickness. On the contrary, IgM and IgG are seen in the blood approximately five and more eight days after the rash's onset, respectively. If IgM and IgG antibodies are identified, an uninfected individual with a history of inflammation and severe illness may have an indirect diagnosis of MPXV. None of these assays, however, is specific to MPX and may detect the presence of other OPV species [63]. Individuals immunized with smallpox may use IgM to determine the presence of MPX infection. A positive IgM capture ELISA shows recent exposure to OPV in both unvaccinated and vaccinated individuals. On the contrary, positive IgG capture ELISA implies exposure to OPV by vaccination or spontaneous infection [64]. Consequently, the presence of both IgM and IgG in the sample provides a strong indication of recent exposure to OPV in individuals who have been previously immunized or were naturally exposed to the virus. Due to the serological cross-reactivity of OPV, antigen and antibody detection methods cannot confirm MPX. Therefore, serology and antigen detection methods are not recommended for diagnosis or investigation when resources are limited [65].

Molecular Method

This involves the use of polymerase chain reaction (PCR) or real-time PCR (RT-PCR), and it is recommended that this test be conducted in a facility with biosafety level three [66]. It uses RT-PCR targeting conserved parts of the extracellular envelope protein gene (B6R) and DNA polymerase gene, E9L; MPXV DNA is commonly found in clinical and veterinary materials and in MPXV-infected cell cultures [67]. MPXV DNA is identified by restriction-length fragment polymorphism (RFLP) of PCR-amplified genes or gene fragments, although RFLP is time-consuming and requires viral culture [68]. Whole genome sequencing using next-generation sequencing (NGS) technology remains the gold standard for characterizing MPXV and other OPV. However, the method is expensive, and sequencing data processing requires enormous computer resources [69]. Consequently, there are better techniques for the characterization of NGS, especially in resource-poor sub-Saharan Africa. Although RT-PCR remains the primary method for routine MPXV diagnosis, it must be augmented with technologies for field genome sequencing. In resource-limited regions of Africa, MinION field sequencing has been efficiently deployed for genomic surveillance of the Ebola outbreak [70].

Phenotype Method

According to clinical diagnosis, the incubation period for MPXV is 4 to 21 days. It is commonly preceded by prodromal disease characterized by headache, fever, back pain, intense asthenia, pharyngitis, sweating, and malaise [71]. Following the prodromal phase, the exanthema phase is marked by vesiculopustular rashes that begin on the face and extend to the rest of the body within 1 to 10 days. Similar to smallpox, lesions in patients with MPXV are monomorphic, pea-sized, and challenging [8,71]. The crop-like appearance and absence of rapid centrifugal spread distinguish it from smallpox. The presence of lymphadenopathy separates MPXV from smallpox according to its clinical manifestations. The cohort of 645 patients determined that the clinical case definition MPX in the absence of laboratory confirmation had high sensitivity (93-98%) but low specificity (9%-26%). However, it is crucial to detect suspected cases during monitoring [72,73].

Histological Method

Histologically, papular lesions show acanthosis, individual keratinocyte necrosis, and basal vacuolization. This is followed by a superficial and deep perivascular lymphohistiocytic infiltrate in the dermis. In vesicular lesions, spongia with reticular and ballooning degeneration is seen [74]. It is possible to see large epithelial cells with many nuclei. Pustular lesions are characterized by epidermal necrosis, an excess of eosinophils and neutrophils, and karyorrhexis in most cells. Necrosis extends the entire thickness of the epidermis, with a distinct lateral demarcation from the adjacent intact epidermis. Petechial lesions indicate subsequent vasculitis. Keratinocytes may include intranuclear amphophilic structures that resemble viral inclusions [74].

MPX prognosis

The incubation period of MPX is usually 6 to 13 days after exposure but ranges from 5 to 21 days. The severity of infection varies considerably; there are two distinct clades of MPXV. With a death rate of less than 1%, West African clades have a better outlook. On other hand, the central basin clade is more dangerous. Up to 1% of unvaccinated children die from it. Unvaccinated individuals and immunocompromised more likely to develop fatal infections [46,75].

MPX management

In African cases, the death rate was between 1% and 10%, and death was linked to the patient's health and other conditions. Most people died from diseases that could have been avoided. No one was killed in the new flare-up in the United States. During the fever phase of illness, people often don't feel well, so they may need to stay in bed and be closely watched. In more severe cases, it may be necessary to stay in the hospital. Medical workers and people close to them should take airborne and contact safety measures to avoid getting sick. Bringing in exciting animals as pets poses a risk to both the health of people and animals because it introduces microbes that are not native to the area. Animals with signs of respiratory trouble, mucocutaneous sores, rhinorrhea, eye discharge, or possibly lymphadenopathy, especially those infected above or in close contact with them, should be kept away from other animals immediately. It is essential to stay away from contact, especially bites, scratches, and liquids. In hospitals, patients are placed in rooms with low air pressure, and healthcare workers are careful with contact and droplets [76,77].

MPX monitoring

Healthcare interventions are required to stop the current outbreak. These include strict isolation and care for case patients while they are still contagious or until the skin lesions dry out and crust. This could take two to four weeks. If available, vaccines may be an excellent addition to this strategy but are not a replacement. Based on the risks and benefits that have been looked at so far and regardless of how many vaccines are available, mass vaccination against MPX is neither necessary nor recommended [78]. To improve prevention and reduce the spread of MPXV, it is also essential to raise awareness among the general public and people who have contact with infected people. So different kinds of educational campaigns can be run. The public and highly vulnerable groups should be educated more about symptoms, signs, how it spreads, and how to stop it. They should also be encouraged to stick to the different ways of preventing it and get medical help [77].

MPXV vaccination

The vaccinia vaccine used to eliminate smallpox is also effective against MPXV. Because both smallpox and MPXV belong to the same genus [78,79]. Recently, ACAM 2000 and JYNNEOS were approved as vaccinations to prevent smallpox. Only JYNNEOS was approved for the prevention of MPXV [80]. JYNNEOS is a live virus vaccination containing Modified Vaccinia Ankara-Bavarian Nordic (MVA-BN), a non-replicating virus. JYNNEOS is used on people ≥ 18 and safely used in significantly immunocompromised individuals. There are not enough human data on JYNNEOS given to pregnant women to know what risks are linked to the vaccine during pregnancy [81]. JYNNEOS is not licensed for people under 18 and has not been thoroughly tested in this group. Clinical studies haven't found that JYNNEOS patients have a higher risk of getting myopericarditis [80].

MPX treatment

The care of MPX is fully optimized to help relieve symptoms, prevent complications, and stop long-term effects. Patients should receive fluids to keep their nutritional status at a reasonable level. Patients who have bacterial infections should be treated [80]. The MPX and smallpox viruses are genetically similar. Antiviral drugs and vaccines to protect against smallpox also prevent and treat MPX infections [82].

Tecovirimat is an antiviral drug approved for treating smallpox and licensed by European Medicines Agency for treating MPX [83]. Until now, tecovirimat was for severe MPX or a high risk of getting severe disease. This includes people with weak immune systems. Tecovirimat might help stop or reduce severe MPX that affects the eyes, throat, genitalia, and anus. It might help with short-term problems like pain, inflammation, abscesses, and scarring. It is taken orally twice a day for two weeks or intravenously [83,84].

Brincidofovir is approved as a treatment for smallpox. It is effective against poxviruses and other double-stranded DNA viruses. It stops the MPXV from spreading by stopping the polymerase-mediated synthesis of DNA. It is given to patients in two doses. Due to the risk of harming the embryo and fetus, brincidofovir is not recommended for pregnant women. People who could have children should not try to get pregnant and use effective birth control during treatment and for at least two months after the last dose [85,86].

Cidofovir is used to treat cytomegalovirus and stop MPXV from spreading by stopping DNA polymerase. It is effective against poxviruses [85,86].

MPX recommendations

Pregnant women exposed to MPX should receive the smallpox vaccine as prophylaxis. Babies born to mothers with MPX are monitored for signs of possible exposure or infection before or during birth [81]. Close contact also put mothers and babies or young children at risk. Children exposed to MPX should be fully vaccinated for their age according to the routine national immunization schedule and have their vaccinations as up-to-date as possible. Children shouldn't sleep in the same room or bed as someone with MPX or use identical cups or plates [81]. Sexually active people should be told to stop having sex until skin sores from MPX have crusted over and a new layer of skin has formed underneath [87-89].

## Conclusions

HMPX is becoming a global issue after formerly being limited to Africa. MPXV spreads from animals to people through infected animals, and it can also spread from person to person, especially among men who have sex with other men. The clinical manifestations of MPX are similar to those of smallpox. Clinical symptoms don't confirm MPXV. Laboratory methods, such as microscopy, immunology, histology, and molecular approaches, should be used for confirmation. The vaccinia vaccine is effective against MPXV. JYNNEOS was approved as a vaccine for the prevention of MPXV. Tecovirimat is licensed to treat people with severe MPX or at a high risk of getting severe disease. We should encourage international cooperation to conduct clinical trials investigating the effectiveness and safety of MPX vaccines and antiviral medications. Precautions must be taken to prevent an MPXV outbreak.
